# *In vivo* antiplasmodial and toxicological effect of *Maytenus senegalensis* traditionally used in the treatment of malaria in Tanzania

**DOI:** 10.1186/s12936-014-0525-y

**Published:** 2015-02-14

**Authors:** Hamisi M Malebo, Victor Wiketye, Shaaban J Katani, Nteghenjwa A Kitufe, Vitus A Nyigo, Calister P Imeda, John W Ogondiek, Richard Sunguruma, Paulo P Mhame, Julius J Massaga, Bertha Mammuya, Kesheni P Senkoro, Susan F Rumisha, Mwelecele N Malecela, Andrew Y Kitua

**Affiliations:** Department of Traditional Medicine Research, National Institute for Medical Research, 3 Barack Obama Drive, P.O. Box 9653, 11101 Dar es Salaam, Tanzania; Ngongongare Medical Research Centre, P.O Box 514, Usa River, Arusha, Tanzania; Traditional Medicine Unit, Ministry of Health and Social Welfare, P.O. Box 9083, Dar es Salaam, Tanzania; Headquarters, National Institute for Medical Research, 3 Barack Obama Drive, P.O. Box 9653, 11101 Dar es Salaam, Tanzania; Government Chemist Laboratory Agency, P. O. Box 164, Dar es Salaam, Tanzania

**Keywords:** *Maytenus senegalensis*, Malaria, *Plasmodium berghei*, Antiplasmodial, Acute toxicity

## Abstract

**Background:**

In Tanzania and elsewhere, medicinal plants, including *Maytenus senegalensis,* are still widely used in the treatment of malaria and other ailments. The aim of the present study was to investigate the *in vivo* antiplasmodial and toxic effects in mice.

**Methods:**

Oral antiplasmodial and acute toxicity of the ethanolic root extract of *M. senegalensis* was evaluated in mice. The Peters 4-day *in vivo* antiplasmodial effect against early rodent malaria infection in chloroquine-sensitive *Plasmodium berghei* NK 65 strain in mice.

**Results:**

The *M. senegalensis* extract was found non-toxic and the oral median lethal dose in mice was determined to be greater than 1,600 mg/kg body weight. The findings revealed a significant (*P* = 0.001) daily increase in the level of parasitaemia in the parasitized untreated groups and a significant (*P* < 0.001) dose dependent decrease in parasitaemia in the parasitized groups treated with varying doses ranging from 25 to 100 mg/kg body weight of *M. senegalensis* extract and the standard drug sulphadoxine/pyrimethamine at 25/1.25 mg/kg body weight. Overall, the dose dependent parasitaemia suppression effects were in the order of: 25/1.25 mg/kg body weight of sulphadoxine/pyrimethamine > 100 mg/kg > 75 mg/kg > 50 mg/kg > 25 mg/kg body weight of *M. senegalensis* extract.

**Conclusion:**

The implications of these findings is that *M. senegalensis* ethanolic root bark extract possess potent antiplasmodial effect and may, therefore, serve as potential sources of safe, effective and affordable anti-malarial drugs. The displayed high *in vivo* antiplasmodial activity and lack of toxic effect render *M. senegalensis* a candidate for the bioassay-guided isolation of compounds which could develop into new lead structures and candidates for drug development programmes against human malaria.

## Background

*Maytenus senegalensis*, which belong to the Celastraceae family, is an abundant perennial tree that has a wider distribution in Africa, Arabia, Afghanistan and India [1]. The decoction of the stem bark and root is used traditionally in the folk medicine in Africa for the treatment of a number of diseases and health conditions, including malaria, fever, chest pains, rheumatism, dysmenorrhoea, diarrhoea, dyspepsia eye infection, wounds and snakebites [2,3].

In Kagera region, in Tanzania, the root bark is used in traditional medicine by traditional healers and the community to treat malaria, fever, pain and chronic illnesses [2,4]. The same traditional uses of *M. senegalensis* are also reported from other African countries namely; Benin, Côte d’Ivoire, Kenya, Senegal, Sudan, Zambia, and Zimbabwe [5-11]. Most of the population in rural communities in Tanzania and in other African countries relies on herbal medicines for their health care needs [12]. This stems from the cultural significance of indigenous people as well as the herbal medicines are generally more accessible, affordable and the perceived efficacy and safety of the remedy [13]. Despite the efforts of government of the United Republic of Tanzania to make modern health service accessible, available and acceptable to all, most of people in rural areas reside distances away from the facilities and that, the road infrastructure linking some of the communities to the health facilities are in some seasons of the year are inaccessible, especially during rainy seasons [14-16].

On this background coupled with other factors make it difficult for the majority of rural dwellers to access quality health care and unequivocally make traditional medicine an obvious choice for them. In Kagera region in Tanzania, dependency on traditional medicine as the first option for rural dwellers in the treatment of malaria has been inevitable. Despite the popular use of *M. senegalensis* for malaria and other ailments, few pharmacological studies have been described in literature and there is no extensive research on the toxicity of extracts of the root bark and their pharmacological effect on malaria. Thus, the interest in this plant was justified by its potential medicinal value against malaria. Therefore, the aim of this study was to investigate the *in vivo* efficacy and safety of the root bark extract of *M. senegalensis*.

## Methods

### Chemicals

Analar grade ethanol and dimethyl sulphoxide (DMSO) were purchased from VWR (UK).

### Plant collection and sample preparation

Root bark materials of *M. senegalensis* were collected by the help of the traditional healers and community members at Kyamlaire village in Kagera region, Tanzania. Identification of the plant species was done at the Herbarium of the Department of Botany, University of Dar es Salaam where a voucher specimen has been deposited (Suleman collection No. 4998). The root bark was air dried, grinded and phytochemical processing to get crude ethanolic extract was done. Two kilograms of air-dried and ground root bark of *M. senegalensis* was soaked in 6 L ethanol for 48 hours at room temperature (about 30°C). The crude extract was obtained by filtration, followed by evaporation of the solvent *in vacuo* at 30°C so as to avoid decomposition of thermally labile compounds. The afforded extract weighing 244 g (12.2% yield) was kept at 4°C in the refrigerator till when needed for assay.

### Animals

Swiss albino mice weighing 18–22 g and aged 6–8 weeks raised at the Animal House of the Government Chemist Laboratory Agency (GCLA) were used in the study. The mice were maintained on standard feed and water (*ad libitum*). Approval for the study was obtained from the Ethical Committee, National Institute for Medical Research (NIMR) in Tanzania.

### Parasite

Blood-stage samples of *Plasmodium berghei* ANKA 65 strain were stored in liquid nitrogen in solution for cryopreservation of the parasite till needed for the assay.

### Infection of mice

Albino mice between 6 to 8 weeks of age and weighing between 18 to 22 grams were randomized into six groups of five each and with the exception of the healthy control group, all mice were inoculated intravenously in the tail vein on day 1 with 1x10^7^*P. berghei* ANKA 65 parasitized erythrocytes obtained by suitable dilution with 0.9% NaCl of infected red blood cells from a sacrificed mouse with 20% parasitaemia.

### Drug administration

This was the four-day suppressive test [17,18]. The *M. senegalensis* root bark ethanolic extract was dissolved in DMSO and then diluted with distilled water to 1% DMSO to make concentrations of 25, 50, 75 and 100 mg/kg body weight in each 0.4 ml of the extract once a day (at 24-hour interval) for four days post-infection (p.i.). Likewise sulphadoxine/pyrimethamine (SP) was dissolved in 1% DMSO at a concentration of 25/1.25 mg/kg in 0.4 ml. The first four groups each were treated with crude *M. senegalensis* root bark ethanolic extract once daily starting on day 1 with 0.4 ml of each one of the test dosage through the oral route. The fifth group was treated with standard drug (SP) and served as the positive control. The sixth group received the vehicle and served as untreated controls. Treatments were continued for days 2, 3, and 4. The course of infection was followed up on day 7 by a Giemsa-stained blood smear determination of parasitaemia.

The percentage of parasitaemia for each group was calculated using the formula:

$$ =\frac{\left(\mathrm{mean}\ \mathrm{parasitaemia}\ \mathrm{treated}\right)\times 100}{\mathrm{Mean}\ \mathrm{parasitaemia}\ \mathrm{control}} $$

The percent inhibition of parasitaemia for each group was calculated using the formula

$$ \begin{array}{l}=100\ \hbox{--} \frac{\left(\mathrm{mean}\ \mathrm{parasitaemia}\ \mathrm{treated}\times 100\right)\kern0.5em }{\mathrm{Mean}\ \mathrm{parasitaemia}\ \mathrm{control}}\\ {}\kern0.5em \end{array} $$

### In vivo acute toxicity assessment

Crude *M. senegalensis* root bark ethanolic extract was assessed for its toxicity *in vivo* in albino mice according to WHO guidelines [19]. Crude *M. senegalensis* root bark ethanolic extract was dissolved in DMSO and then diluted with distilled water to 1% DMSO to make concentrations of 200, 300, 400, 800 and 1600 mg/kg body weight. Five groups of ten albino mice each were used during the study including five males and five females. Male and female young adult mice were randomized and doses of *M. senegalensis* were administered by oral gavage. After drug administration, food was withheld for 2 h. Mice were observed individually for 4 hours after drug administration and then at the 24 h and 48 h. All mice were observed to identify any symptoms manifesting ill-health or behavioural changes. Visual observation parameters included alertness, grooming, tremors, convulsions, urination, salivation and writhing. The median oral lethal dose calculated as the geometric mean of doses that caused between 0 and 100% mortality (if any). The definition of the acute toxicity used was: 0 ≤ 50 mg/kg body weight – highly toxic; 50 ≤ 300 mg/kg body weight - toxic; 300 ≤ 1,000 mg/kg body weight - moderately toxic; 1,000 ≤ 2,000 mg/kg body weight – mildly toxic; and 2,000 ≤ 5,000 mg/kg body weight – non-toxic.

### Statistical analysis

Data was analysed by statistical software SPSS version 11.5 (SPSS Inc, Chicago, Illinois, USA). Numerical data presented as mean ± standard deviation, the significance of mean difference between two independent groups was determined by using Student’s *t*-test, and one-way analysis of variance (ANOVA). A *P* value <0.05 was considered significant. Proportional data was presented as percentages. Significance testing of differences between proportions was done by using Fisher’s exact test, a *P* value <0.05 was considered significant.

## Results

### *In vivo* anti-plasmodial activity

**Figure 1:**
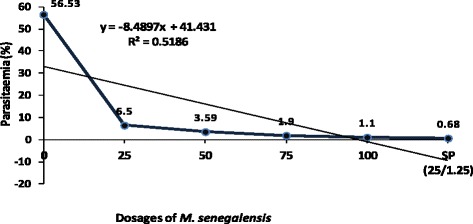
**Suppression of** ***Plasmodium berghei*** **parasitaemia exhibited by different dosages of** ***M. senegalensis*** **in comparison to controls in experimentally infected mice.**

**Figure 2:**
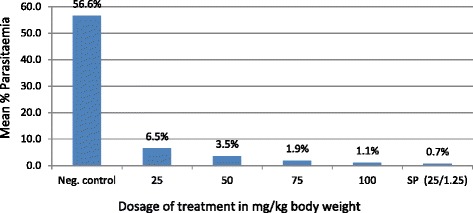
**Percent parasitaemia observed with different dosages of** ***M. senegalensis*** **in comparison to controls in experimentally infected mice.**

The results of the *in vivo* antiplasmodial effect of *M. senegalensis* ethanol extract showed a dose-dependent significant decrease in malaria parasite count in mice infected with *P. berghei* (*P* = 0.001). Table 1 and Figures 1 and 2 show summary of the percentage parasitaemia in mice on day 7 post-infection (p.i.).

**Table 1:** **Percent Parasitaemia per dose in mg/kg body weight of mouse**

**Doses**	**Percent Parasitaemia per mouse (%)**					**Mean (%)**	***P*** **value of Tests vs Neg. control**
	**1**	**2**	**3**	**4**	**5**		
25	6.25	6.75	6.50	6.80	6.20	**6.50**	P < 0.001
50	3.66	3.69	3.43	3.80	3.40	**3.59**	P < 0.001
75	2.06	2.10	1.54	2.00	1.80	**1.90**	P < 0.001
100	0.90	0.96	1.16	1.08	0.90	**1.10**	P < 0.001
Neg. control	56.58	56.45	56.57	56.56	56.51	**56.53**	
SP (25/1.25)	0.49	0.80	0.76	0.72	0.65	**0.68**

**Table 2:** **The calculated percentage of suppression (inhibition) of** ***Plasmodium berghei*** **per dose**

**Dose in mg/kg body weight**	**Calculation of % inhibition of parasitaemia**	**Percent inhibition**	***P*** **value of Pos. control vs Tests**
25	100 - [6.5/56.53 × 100]	**88.50**	P < 0.05
50	100 - [3.59/56.53 × 100]	**93.65**	P < 0.05
75	100 - [1.9/56.53 × 100]	**96.64**	P > 0.05*
100	100 - [1.1/56.53 × 100]	**98.054**	P > 0.05*
Negative control	100 - [56.53/56.53 × 100]	**0**	
Positive control (SP) (25/1.25	100 - [0.52/56.53 × 100]	**99.08**	

*SP (standard drug) has no marked superiority to M. senegalensis extract at a dose of 75-100 mg/kg.

**Table 3:** **Probit analysis of** ***in vivo*** **antiplasmodial activity of** ***M. senegalensis*** **ethanol extract in experimental mice**

**Probit**	**Antiplasmodial activity in mg/kg BDW**	**Range at confidence interval 95% limits (mg/kg BDW)**
ED_50_	3.3	1.8 to 4.9
ED_75_	10.2	7.2 to 13.5
ED_90_	28.4	21.6 to 39.3
ED_99_	166.5	102.4 to 349.8

Slope = 1.361 + −0.162; Natural Response. = 0.000 + −0.000; Heterogeneity = 0.04.

**Figure 3:**
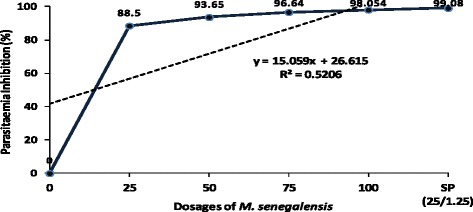
**Suppression of** ***Plasmodium berghei*** **parasitaemia exhibited by different dosages of** ***M. senegalensis*** **in comparison to controls in experimentally infected mice.**

Based on day 7 p.i. smears, the *M. senegalensis* ethanol extract dosages ranging from 25-100 mg/kg body weight, exhibited significant suppression of parasitaemia ranging from 88.5% to 98.1% (*p* = 0.001) as shown in Table 2 and Figure 3. The highest reduction of parasitaemia was observed at a dose of 100 mg/kg body weight of mice treated with *M. senegalensis* ethanol extract as compared to the negative control group. At 100 mg/kg body weight, the *M. senegalensis* ethanol extract exhibited 98.1% suppression of parasitaemia comparable to 99.1% that of SP, the positive control. The *in vivo* antiplasmodial activity of *M. senegalensis* was expressed by the dose inhibiting 50%, 75%, 90% and 99% of parasite growth (ED_50_, ED_75_, ED_90_, ED_99_). The effective dose that cured 50% of test mice (ED_50_) was calculated as 3.3 mg/kg body weight which indicates that the *M. senegalensis* ethanol extract has high *in vivo* antiplasmodial activity in mice infected by *P. berghei* (Table 3). The extract also revealed other promising effective doses (ED_75_, ED_90_ and ED_99_) at calculated dosages of 10.2, 28.4 and 166.5 mg/kg body weight, respectively.

**Table 4:** ***In vivo*** **acute toxicity of** ***M. senegalensis*** **ethanol extract in experimental mice**

**Dose in mg/kg body weight**	**Acute toxicity (survival at 24 and 48 hours)**		
	**No. of mice**	**24 hours**	**48 hours**
200	10	10	10
300	10	10	10
400	10	10	10
800	10	10	10
1600	10	10	10

### Acute toxicity

Results of the acute toxicity evaluation of *M. senegalensis* ethanol extract are provided in Table 4. At all the tested dosages no any mouse died at 24 and 48 hours post drug administration. The highest tested dose was 1600 mg/kg of body weight. The animals were not exposed to doses higher than 1,600 mg/kg of body weight as with increasing concentration, the extract precipitated and the solution became too sticky to handle. Observations of physical conditions, toxic symptoms showed that no any sign of toxicity was seen in experimental mice at the tested dosages. The therapeutic index (TI) calculated as ratio of the LD_50_ and ED_50_ was estimated to be higher than 113.5, indicating that *M. senegalensis* ethanol extract is not toxic.

**Table 5:** **Toxicity effect manifestation of** ***M. senegalensis*** **ethanol extract in experimental mice**

**Response**	**Dosage of extract administered to test mice (mg/kg body weight)**									
	**200**		**300**		**400**		**800**		**1600**	
	**Before**	**After**	**Before**	**After**	**Before**	**After**	**Before**	**After**	**Before**	**After**
1. Alertness	N	N	N	N	N	N	N	N	N	N
2. Grooming	A	A	A	A	A	A	A	A	A	A
3. Tremors	A	A	A	A	A	A	A	A	A	A
4. Convulsion	A	A	A	A	A	A	A	A	A	A
5. Urination	N	N	N	N	N	N	N	N	N	N
6. Salivation	N	N	N	N	N	N	N	N	N	N
7. Writhing	A	A	A	A	A	A	A	A	A	A

Key: N = normal; A = absent.

Table 4 shows the toxicity manifestation parameters observed before and after the administration of *M. senegalensis* ethanol extract. The statistical analysis of the dosage administered to mice and the observed effects were found significant at 5%. No any toxic effect manifestation was observed even at the highest dosage of 1600 mg/kg body weight of the test mouse (Table 5). This clearly confirms that, the *M. senegalensis* ethanol extract do not produce oral toxicity at the tested dosages.

The lethal dose of *M. senegalensis* ethanol extract is higher than 1,600 mg/kg body weight and hence, in a single dose administration, the plant extract caused no any mortality.

## Discussion

The ethanolic root bark extract of *M. senegalensis* showed high antiplasmodial activity against *P. berghei* infection in mice as evidenced by the percentage of inhibition of parasite development. The *M. senegalensis* extract exhibited higher suppression of malaria parasites comparable to that of the standard antimalarial drug, SP. *Maytenus senegalensis* extract exhibited a dose dependent activity, as the dose increased also antiplasmodial activity increased significantly. In addition, the observed chemosuppressive activity suggests that the root bark extract of this plant can suppress parasite growth to lower levels of parasitaemia in a long-term administration as it is practiced in traditional medicine. However, findings showing lower antiplasmodial effect with parasitaemia suppression of 7% and 23% of *M. senegalensis* leaves and root bark, respectively, have been reported [10]. It was furthermore revealed that, the parasitaemia suppression of *M. senegalensis* leaves and root bark extracts were potentiated by chloroquine to 55.4% and 56.2%, respectively [10]. High antiplasmodial effects on parasitaemia in this study are similar to the ones reported by Gessler *et al*. [2], whereby *M. senegalensis* extract at the dosage of 500 mg/kg body weight/day in four days produced a significant reduction of parasitaemia of 90%. Previous *in vitro* screening revealed *M. senegalensis* extract exhibits strong activity against the multi-drug resistant *Plasmodium falciparum* strain K1 with IC_50_ values of ranging from 1.0 to 3.9 μg/ml [2,20,21]. The extracts of *M. senegalensis* also exhibit potent antiinflammatory, analgesic and antipyretic properties in mice [22,23]. These findings support the ethnomedical use of *M. senegalensis* in the treatment of malarial and associated symptoms.

In this study, the extract of *M. senegalensis* did not show any toxic effect because doses up to 1600 mg/kg caused no any death or alter the behaviour of the tested normal mice. It can be extrapolated that, the median lethal dose is thus greater than 1600 mg/kg making *M. senegalensis* safety to be between slightly toxic to non-toxic. However, Da Silva *et al*., in the preliminary toxicity study, noted signs of toxicity of *M. senegalensis* in mice and rats at 1,200 mg/kg body weight [24]. However, a similar finding has not yet been found by other researchers worked on *M. senegalensis* extracts. Recent investigation in Tanzania has further revealed that, *M. senegalensis* extract is practically non-toxic as it is well tolerated and without any sign of toxicity in experimental mice. In the acute toxicity study, it was indicated that, the extract at a dose of 5,000 mg/kg body weight caused neither visible signs of toxicity nor mortality in mice, suggesting its safety [25]. In the classification of toxicity, the extract with an LD_50_ > 5,000 mg/kg body weight *per os* (p.o.) is considered to be non-toxic. Investigation in Mali revealed that, the aqueous extract of *M. senegalensis* administrated orally in mice caused no any toxicity to mice [22]. Previous findings also suggest that *M. senegalensis* is practically non-cytotoxic as it exhibited IC_50_ values of 87.82 ± 3.02 and >90.00 against mammalian cell lines *viz*; Vero cell lines and Rat skeletal myoblast (L-6) cells, respectively [20,21]. The oral non-toxic nature of *M. senegalensis* and the use of this plant against malaria and other ailments go hand in hand with scientific evidence provided by this study.

## Conclusion

The oral administration of graded *M. senegalensis* ethanol extract doses (25–100 mg/ kg body weight) to mice for 4 days significantly suppressed malaria parasites development *in vivo* against *Plasmodium berghei* NK 65 strain in experimental mice. Higher doses of *M. senegalensis* ethanol extract (200–1600 mg/ kg body weight) could not result in death and was not associated with adverse effects. According to these results, the *M. senegalensis* ethanol extract could be categorized as a non-toxic crude drug that acts harmlessly under the normal traditional usage. The observed large safety window of *M. senegalensis* extract is responsible for its widespread use in Tanzania and other countries without acute toxic symptoms and complications. Hence, *M. senegalensis* may be exploited as herbal pharmaceuticals for malaria, fever and pains. The implications of these findings is that *M. senegalensis* ethanolic root bark extract possess potent antiplasmodial effect and may therefore serve as potential sources of safe, effective and affordable antimalarial drugs. The displayed high *in vivo* antiplasmodial activity and lack of toxic effect render *M. senegalensis* a candidate for the bioassay-guided isolation of compounds which could develop into new lead structures and candidates for drug development programmes against human malaria.
